# Carbon Quantum Dots for Zebrafish Fluorescence Imaging

**DOI:** 10.1038/srep11835

**Published:** 2015-07-02

**Authors:** Yan-Fei Kang, Yu-Hao Li, Yang-Wu Fang, Yang Xu, Xiao-Mi Wei, Xue-Bo Yin

**Affiliations:** 1 Research Center for Analytical Sciences, College of Chemistry, Nankai University, Tianjin Key laboratory of Biosensing and Molecular Recognition, State Key Laboratory of Medicinal Chemical Biology, and Collaborative Innovation Center of Chemical Science and Engineering (Tianjin), Tianjin 300071, China; 2 Tianjin Key Laboratory of Tumor Microenviroment and Neurovascular Regulation, School of Medicine, Nankai University, Tianjin, 300071, China

## Abstract

Carbon quantum dots (C-QDs) are becoming a desirable alternative to metal-based QDs and dye probes owing to their high biocompatibility, low toxicity, ease of preparation, and unique photophysical properties. Herein, we describe fluorescence bioimaging of zebrafish using C-QDs as probe in terms of the preparation of C-QDs, zebrafish husbandry, embryo harvesting, and introduction of C-QDs into embryos and larvae by soaking and microinjection. The multicolor of C-QDs was validated with their imaging for zebrafish embryo. The distribution of C-QDs in zebrafish embryos and larvae were successfully observed from their fluorescence emission. the bio-toxicity of C-QDs was tested with zebrafish as model and C-QDs do not interfere to the development of zebrafish embryo. All of the results confirmed the high biocompatibility and low toxicity of C-QDs as imaging probe. The absorption, distribution, metabolism and excretion route (ADME) of C-QDs in zebrafish was revealed by their distribution. Our work provides the useful information for the researchers interested in studying with zebrafish as a model and the applications of C-QDs. The operations related zebrafish are suitable for the study of the toxicity, adverse effects, transport, and biocompatibility of nanomaterials as well as for drug screening with zebrafish as model.

## Carbon quantum dots (C-QDs)

Nanomaterials provide much appreciable opportunities for bioimaging and clinical diagnosis^1^. Semiconductor quantum dots (QDs) are molecular-sized nanocrystals that are less than 10 nm in size. Due to their strong quantum confinement effects, QDs exhibit several unique photoelectric properties, such as size- and wavelength-dependent luminescence and low photo-bleaching^2^,^3^,^4^. QDs-based imaging integrates these properties to improve fluorescence imaging with the merits including real-time, accurate, and *in vivo* observations with high sensitivity, rapid responses, low costs, and no radiation. However, metal-based QDs (M-QDs) are associated with long-term toxicity and environmental concerns owing to the use of heavy metals^5^,^6^. Complex procedures are required to obtain M-QDs, and their aggregation is often observed after long-term storage^7^.

Carbon QDs (C-QDs) are a type of quasi-spherical carbon material with diameters less than 10 nm^8^. C-QDs do not have the toxic effect observed from heavy metals because C-QDs contain large amounts of carbon and relatively low amounts of oxygen, hydrogen, and nitrogen^9^,^10^. While carbon exists in the form of crystalline graphite as the core of C-QDs, oxygen and hydrogen forms hydroxy and carboxyl groups, which facilitate the functionalization and improve the hydrophilicity of C-QDs^8^. C-QDs also exhibit the wavelength-dependent luminescence and low photo-bleaching. Moreover, C-QDs have good storage- and photo-stability^11^. Favorable stability of the fluorescence intensity of C-QDs was observed after storage for 1 year under ambient conditions^12^. Thus, C-QDs are becoming a desirable alternative to M-QDs when taking their low toxicity, unique photophysical and chemical properties into considerations.

C-QDs have been prepared from graphite^13^, soot^14^, and carbonized products through chemical oxidation with strong acids or oxidants in the top-down procedure^8^,^15^. The bottom-up approach is diverse because various molecular precursors can be hydrothermally treated to obtain C-QDs^8^. While strong acids and oxidants are not required, passivated agents provide the possibility to obtain surface-passviated and/or heteroatom-doped C-QDs with improved luminescence^8^. A C-QD yield of 78% was obtained using ethylenediamine tetraaceticacid (EDTA) as a precursor^16^. Glucose, in combination with various passivated agents, has been extensively used to prepare C-QDs with enhanced fluorescence, high yield, simple procedure, low cost, and high biocompatibility^17^,^18^. Here, we provided the details for preparing C-QDs with glucose and ethylenediamine as the precursor and passivated agent^19^,^20^,^21^,^22^,^23^ and their imaging applications in an important model organism, zebrafish.

## Zebrafish

Zebrafish are important model organisms because of their rapid embryonic development and short generation time^24^. The timing of embryo laying can be controlled by regulating the light−dark cycle^25^. Caring for zebrafish and embryos is also simple. Zebrafish have been used for *in vivo* imaging, behavior testing, and compound screening^26^,^27^. A special zebrafish edition of *Methods* was released in 2013 within three areas to discuss the new technologies available for using zabrafish, including genomics and epigenomics, regenerative medicine and modeling human disease, and imaging advances related to zebrafish^27^. Importantly, zebrafish are highly homologous with mammals and approximately 70% of human genes are orthologous to those in zebrafish^28^,^29^, so modeling human disease with zebrafish as model becomes an important and fast strategy. As an important milestone for its use as a model organism, the zebrafish genome was reported in 2013^28^,^29^.

Embryonic development is an important model for studying the toxicity, *in vivo* system damage, and the transport and biocompatibility of nanoparticles and drugs^3^,^30^. The possible risks posed to human health and the environment are proposed based on these developmental results. Zebrafish embryo is unique for these studies compared with other vertebrate model systems such as mice and rats^31^,^32^. The embryonic development of zebrafish is rapidly completed within 120 h and their developmental stages are well-characterized. The particular external development of zebrafish embryos facilitates direct visual detection of pathological embryonic death and maldeveloped phenotypes. Moreover, zebrafish embryos are transparent and it is easy to obsvere the transport and the effects of nanoparticles as well as conduct drug screening in real-time^31^,^32^. Dose-dependent biocompatibility, the initial entry steps into embryos, and the potential applications and adverse effects of nanomaterials and drug screening have been demonstrated based on the embryo development results^3^,^33^,^34^. Introducing extraneous species into the embryos is critical for these types of studies.

Zebrafish has already been widely used in biological and biochemical research, such as fluorescent protein expression^35^, visualizing blood flow in the heart *in vivo*^36^, gene knockdown and functional cardiac imaging^37^, studying mutants with defects in bone calcification^38^, and assessing the oxidative toxic effects of nanoparticles^39^. Primordial germ cells (PGCs) of zebrafish were monitored to examine their morphology during early development^40^. Zebrafish embryogenesis has been extensively studied by *in vivo* imaging^41^. Here, we describe zebrafish culture and the operations involved in zebrafish fluorescence imaging using C-QDs as a probe. Introducing C-QDs into zebrafish embryos and larvae by microinjection and soaking was illustrated in detail.

C-QDs can enter into the embryos across the chorion and the germ ring in the yolk by simple soaking embryos in C-QDs’ solution. Their distribution in the embryos and larvae of zebrafish are observed from C-QDs’ fluorescence and validates the potential of C-QDs as probe. Microinjection procedures have been used for generating transgenic zebrafish after injecting DNA, for gene knockdown after injecting antisense morpholinos, for studing infectious diseases after injecting microbes, and for studying tumor progression after injecting cancer cells^42^. The procedures used for microinjecting C-QDs into zebrafish embryos were described for fluorescence bioimaging. Microinjection procedure can be adapted to the zebrafish embryo model using other nanomaterials or drugs after some fine adjustment. Zebrafish culture and handling is simple and convenient, while C-QDs are biocompatible and have low toxicity. The fluorescence bioimaging of zebrafish using C-QDs as a probe in this work is an example that be applied to basic life science research and drug screening.

## Results and Discussion

### Characterization of C-QDs

**Figure 1:**
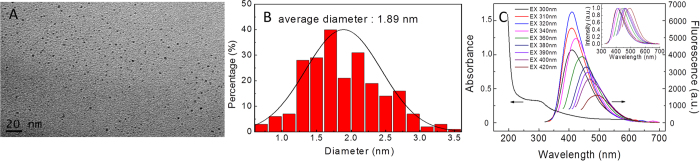
Transmission electron microscopy (TEM) images (**A**)and size distribution (**B**) of C-QDs. (**C**) Absorption and fluorescence spectra of C-QDs using different excitation wavelengths. Inset: the normalized fluorescence spectra. Scale bar, 20 nm.

Since they were first discovered in 2004^43^, C-QDs has been extensively studied for their preparation and photoelectric properties, such as fluorescence, electrochemiluminescence and catalysis^44^. Their excitation-dependent emission characteristic enables C-QDs multicolor cell imaging^44^. Two-photon imaging with C-QDs as a probe was achieved by Sun’s Group^45^. The preparation of C-QDs with glucose as the precursor is relatively simple through a hydrothermal procedure. Moreover, ethylenediamine, as a passivated agent, can enhance the fluorescence emission significantly^46^. Figure 1 shows the transmission electron microscopy (TEM) image and the size distribution of C-QDs prepared using glucose and ethylenediamine as the precursors. C-QD sizes distribute primarily in the range of 1–3 nm with an average diameter of 1.89 nm (Fig. 1B), confirming the nanoscale of C-QDs for their quantum confinement effect. The absorption and fluorescence spectra of C-QDs under different excitation wavelengths are shown in Fig. 1C. The normalized emission spectra of C-QDs in the inset of Fig. 1C clearly illustrate excitation-dependent emission, which provides the possibility of multicolor imaging with C-QDs as probe.

**Figure 2:**
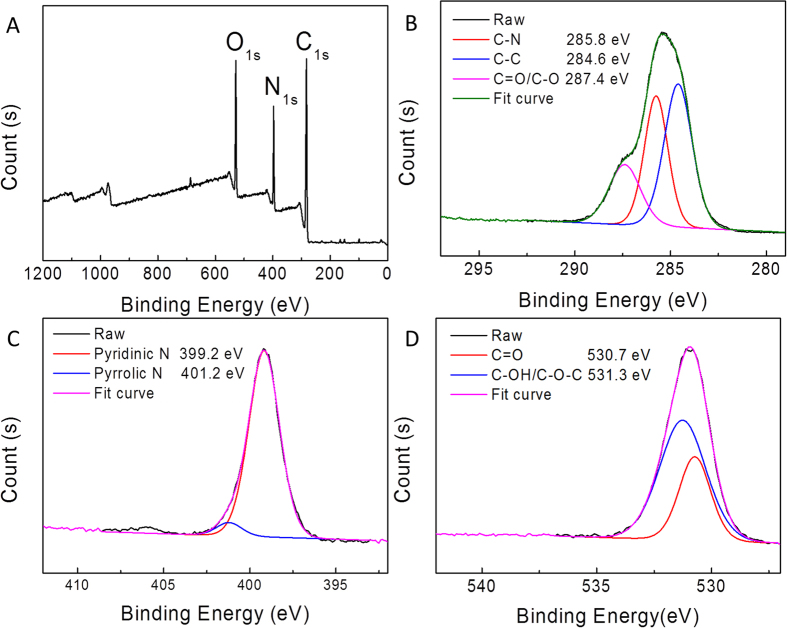
(**A**) XPS spectrum of C-QDs. High-resolution spectra XPS of C1s (**B**), N1s (**C**) and O1s(**D**).

X-ray photoelectron spectroscopy (XPS) analysis shows the composition of as-synthesized C-QDs. C, N, and O were found in the C-QDs from the XPS spectrum as shown in Fig. 2A. The C1s peak was resolved into three components centered at 284.6, 285.8, and 287.4 eV, which represent sp^2^C-sp^2^C, N-sp^2^C and C-O/C = O bonds^21^,^47^,^48^, respectively (Fig. 2B). The high-resolution spectra of N1s (Fig. 2C) revealed the presence of both pyridinic (399.2 eV) and pyrrolic (401.2 eV) N atoms^21^,^48^, illustrating that the C-QDs were successfully doped with nitrogen atoms. Fig. 2C revealed that the O1s peak can be resolved into two components centered at 530.7 and 531.3 eV, representing the presence of the C = O and C-OH/C-O-C groups^49^,^50^. The XPS results indicate that the surface of the as-synthesized C-QDs is functionalized by multiple oxygen- and nitrogen-containing groups by the reaction between glucose and ethylenediamine.

### Imaging of Embryos Introduced C-QDs by Soaking

**Figure 3:**
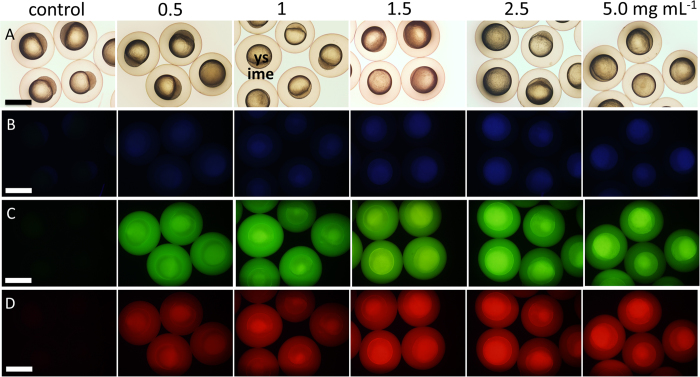
Brightfield (A) and multicolor fluorescence [(B) blue, (C) green, and (D) red] images of zebrafish embryos at 3 hpf after soaking for 3 h in C-QDs solutions with different concentrations. Images acquired under bright light show the yolk sac (ys) and the inner mass of embryos (ime). A 10× ocular lens and 4× objective lens were used. Scale bars, 1.0 mm.

Zebrafish has already been widely used in biological and biochemical research^40^. We selected zebrafish as model because it has been applied extensively in the medical and chemical research. Zebrafish embryos are also an important model for studying toxicity, *in vivo* system damage, and the transport and biocompatibility of nanoparticles and drugs^3^,^30^. Multicolor *in vivo* fluorescence imaging with C-QDs as probe was validated as shown in Fig. 3, which presents brightfield and multicolor fluorescence images of embryos after soaking with C-QDs. The different brightness between the yolk and the inner mass of embryos in fluorescence images show the different affinities of C-QDs to these tissues (Fig. 3B–D). Thus, C-QDs entered into embryos across the chorion and the germ ring by simple soaking and mainly deposited in the yolk sac because of their small size. The fluorescence images of embryos become brighter with the increasing concentrations of C-QDs (Fig. 3B–D), whereas their brightfield images do not differ from each other (Fig. 3A). A blue fluorescence image of embryos was observed using irradiation with ultraviolet light (Fig. 3B), while green and red images are observed when irradiating with blue light and green light, respectively (Fig. 3C,D). C-QDs fluorescence enables the observation of their distribution in the embryos of zebrafish and validates the practicability of C-QDs as imaging probe.

**Figure 4:**
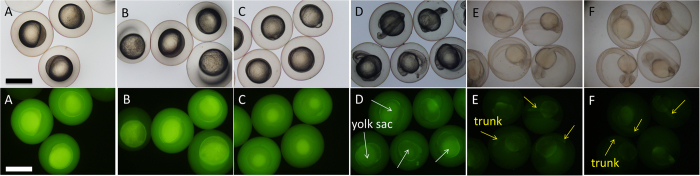
Brightfield (upper) and fluorescence (lower) images of zebrafish embryos after soaking in 2.5 mg mL^−1^ C-QDs solution for 3 h at time points: (**A**) 3, (**B**) 6, (**C**) 12, (**D**) 24, (**E**) 48, (**F**) 60 hpf. A 10× ocular lens and 4 × objective lens were used. Scale bars, 1.0 mm.

The fluorescence imaging of zebrafish embryo after soaking with C-QDs enables the visualization of the embryo development, i.e. from the single-cell to larva (Fig. 4). From the fluorescence images of embryos that soaked in a 2.5 mg mL^−1^ C-QD solution at different period (lower row in Fig. 4D–F), C-QDs were mainly deposited in the yolk sac at 24 hpf (white arrows in Fig. 4D) and redistributed to the trunk of larvae at 48 hpf (yellow arrows in Fig. 4E,F). This redistribution of C-QDs during the embryo development reveals that C-QDs have different tissue affinities. As the incubation time increases, embryos become darker and darker. At 60 hpf, C-QDs fluorescence emission in embryos have nearly disappeared. We assume that some C-QDs are removed from embryos by the digestive system, as shown by the bright gut at 48 hpf (Fig. 4E). By comparing the brightfield images of embryos after cultured in C-QDs solution (upper row in Fig. 4) for 3 h and the control (upper row in Fig. S1) at different periods, we find that C-QDs do not disturb the embryo development, which demonstrate the low toxicity and high biocompatibility of C-QDs. Because of their stable fluorescence emission and biocompatibility, C-QDs were used to reveal the spatial–temporal progression of biological phenomena related to zebrafish embryo development.

### Imaging of Embryos Microinjected with C-QDs

Microinjection of C-QDs was used to illustrate the procedure for direct introduction of extraneous species to zebrafish embryos. The brightfield and fluorescence images of the embryos microinjected with C-QDs are shown in Fig. S2. With increasing C-QD concentration, the fluorescence images of embryos become brighter and brighter (lower row in Fig. S2), while their bright-field images do not differ significantly (upper row in Fig. S2). Embryo brightness indicates that C-QDs have been successfully introduced into embryos by microinjection and the embryos remain alive. Thus, microinjection is a strategy for introducing these materials directly into embryos.

Microinjection procedures have been used for generating transgenic zebrafish after injecting DNA, for gene knockdown after injecting antisense morpholinos, for studying infectious diseases after injecting microbes, and for studying tumor progression after injecting cancer cells^42^. The procedures used for microinjecting C-QDs into zebrafish embryos can also be used for the above study, such as other nanomaterials or drugs with zebrafish embryo as model. Interestingly, the C-QDs have different distribution in zebrafish embryos with different introducation methods: soaking or microinjection. This phenomenon may be crucial for the further medical and chemical application of the C-QDs.

### Imaging of Zebrafish Larvae Introduced C-QDs by Soaking

**Figure 5:**
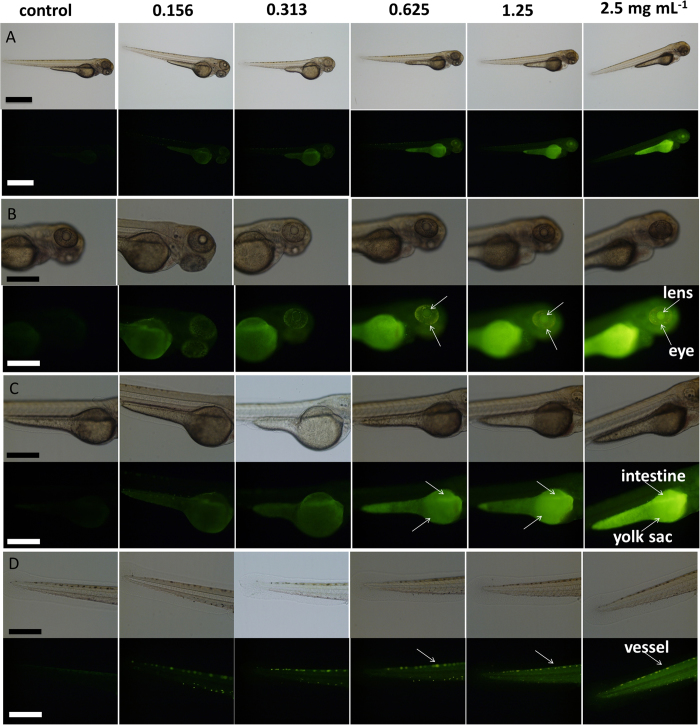
Brightfield (upper) and fluorescence (lower) images of (A) whole bodies, **(B)** head, **(C)** yolk sac, and (D) tail of zebrafish larvae at 84 hpf after soaking for 10 h in C-QDs solution of different concentrations. Enlarged images showing **(B)** eye and lens, **(C)** yolk sac and intestine, as well as **(D)** vessel in tail, with fluorescent images. A 10× ocular lens was used for (**A**, **B**, **C**, and **D**). A 4× objective lens was used for (**A**) and a 10× objective lens was used for (**B**, **C**, and **D**). Scale bars, 1.0 mm for (**A**) and 500 μm for (**B**), (**C**) and (**D**).

Zebrafish larva was used as a model to validate the *in vivo* imaging application and tissue distribution of C-QDs. Figure 5 shows images of whole bodies and the amplified parts of zebrafish larva after soaking in C-QDs solution at different concentrations for 10 h. The zebrafish larvae exposed to the C-QDs became brighter and brighter with increased C-QD concentration in a concentration-dependence mode, illustrating C-QDs were successfully introduced into the larvae. After C-QDs enter into the larvae body through swallowing and skin-absorption^51^,^52^, they accumulate selectively in the head, yolk sac and the tail, showing the tissue-dependent affinity of C-QDs (Fig. 5). The brightness of the dorsal aorta reveals that C-QDs have entered the circulatory system (Fig. 5D), which is important for C-QDs transport in zebrafish. The eyes were the brightest part of the zebrafish head and its brightness increased with C-QDs concentration, and the lens can be readily distinguished from the eyeball (Fig. 5B). This indicates that C-QDs can enter the eye across the blood–ocular barrier. C-QDs in the yolk sac mainly accumulated in the intestine and indicated that C-QDs entered into the digestive system and can be eliminated from the body (Fig. 5C). Some C-QDs were removed from the zebrafish larva by metabolism, which was confirmed by the bright gut (Fig. 5A). The C-QDs preferentially accumulate at the periphery of the zebrafish (Fig. 5B,D), reveals that skin-absorption is one important route for C-QDs entering into zebrafish. The outline of the zebrafish is therefore illustrated by the fluorescence emitted from C-QDs.

The absorption, distribution, metabolism and excretion (ADME) route of C-QDs in zebrafish is therefore revealed by their distribution. C-QDs enter into the body of the zebrafish through swallowing and skin-absorption^52^, and are excreted through the gut partly. Some C-QDs enter into the cardiovascular system and are transferred throughout the whole body, which was confirmed by the brightness in the blood vessel and the tissue in the tail. So, C-QDs are a kind of biocompatible probe without apparent quenching, suitable for *in vivo* imaging. Zebrafish have high homology with mammals^28^,^29^, so the results obtained from zebrafish are therefore used to model biological effects in other higher animals.

**Figure 6:**
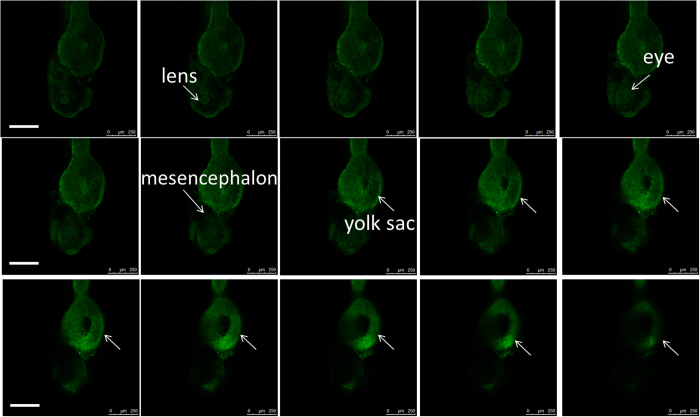
Confocal images of zebrafish (72 hpf larva soaked in 2.50 mg mL^−1^ C-QDs for 10 h) at different scan-planes. The lens, eye, yolk sac and mesencephalon are clearly illustrated. A 10× ocular lens and a 10× objective lens were used. Scale bars, 250 μm.

C-QD fluorescence can also be used to illustrate zebrafish morphology using laser scanning confocal imaging. As shown in Fig. 6, C-QDs accumulate on the yolk sac and the eyeball of the larvae. The confocal images at different scan-planes clearly show the saccate structure of the yolk sac. The C-QDs were also found to accumulate on the eyeball and enter into the lens which means the C-QDs can enter the eye across the blood-ocular barrier. The confocal images results agree well with the results in Fig. 5. The darkness of zebrafish larva mesencephalon indicated that C-QDs cannot cross the blood-brain barrier (BBB) to enter the brain. All of the results confirm that C-QDs have the potential for *in vivo* imaging because they are readily transported through the cardiovascular and digestive systems. Since ocular development and morphology in the zebrafish is similar to that in other vertebrates, the selective accumulation of C-QDs in the eye region could also occur in other vertebrates, indicating the potential imaging applications of C-QDs. Moreover, the dark appearance of the zebrafish head interior indicates that C-QDs cannot cross the BBB to enter the brain. This suggests that C-QDs can be used for eye-related imaging and the potential of cerebral injury would be low.

### Biological toxicity and biocompatibility of C-QDs

Zebrafish embryos and larvae were used as models to validate the *in vivo* biological toxicity and biocompatibility of the as-synthesized C-QDs after soaking/microinjection. As illustrated in the Fig. S3A, the livability of zebrafish embryos is higher than 80% at 24 hpf after microinjected with 1.5 mg mL^−1^ C-QDs solutions during 0 ~ 3 hpf, whereas the livability decreased to about 55% with 2.5 mg mL^−1^ of C-QDs solutions . The livability of embryos soaking in low concentration C-QDs solutions (0.5, 1.0, 1.5 mg mL^−1^) during 0 ~ 3 hpf is higher than 80% at 24 hpf, whereas the livability decreased to about 60% with 2.5 mg mL^−1^ of C-QDs solutions (Fig. S3B). Figure S4 shows the livability of zebrafish larvae at 84 hpf after soaking in C-QDs solutions for 10 h. The livability of larvae soaked in low concentration C-QDs solutions (0.156, 0.313, 0.625 mg mL^−1^) is higher than 95%, similar to that of the control group. After soaking in 1.25 and 2.5 mg mL^−1^ C-QDs solutions for 10 h, the livability of zebrafish larvae are higher than 85% and 55%, respectively. The toxicity of maghemite@SiO_2_ rattle type microspheres were studied with zebrafish as model and 200 μg mL^−1^ of maghemite@SiO_2_ was found to cause severe deformity of zebrafish^53^. Thus, C-QDs showed a lower toxicity than maghemite@SiO_2_ microspheres. Compared with metal-based QDs and other nanoparticles^53^,^54^,^55^,^56^, C-QDs have the higher tolerance concentration by zebrafish embryos and larvea.

Moreover, the zebrafish larvae grow normally after soaking in 1.5 mg mL^−1^ C-QDs solution as shown in Fig. S5. The experiment group had little malformation, almost the same as the control group. The result also confirmed the good biocompatibility of our C-QDs. The C-QDs’ fluorescence in the zebrafish larva became weaker and weaker with increased incubation time (Fig. S5B). The C-QDs in the head and tail are removed more quickly than that in the yolk sac. The intestine (yellow arrows in Fig. S5B) is always the brightest part in the larva, which further confirm the conclusion that the C-QDs are removed from the larva through metabolism. So, C-QDs were removed from the larva and did not affect the development of larva.

## Conclusion

As a conclusion, fluorescence imaging of zebrafish using C-QDs as probe was reported in terms of the preparation of C-QDs, zebrafish husbandry, embryo harvesting, and introduction of C-QDs into embryos and larvae by soaking and microinjection. C-QDs fluorescence enables the observation of their distribution in the embryos and larvae of zebrafish and validates the use of C-QDs as an imaging probe. The properties of C-QDs, such as multi-color, low toxicity, and high biocompatibility were also validated with zebrafish as model. The multi-color fluorescence of C-QDs was still kept in zebrafish embryos and could be used for *in vivo* multi-color imaging. The toxicity, effect on the embryo development, and bio-distribution of C-QDs in zebrafish were clearly illustrated. The C-QDs can enter into the zebrafish embryos and larvae by soaking and concentrate in different part, which reveals their tissue affinity towards zebrafish. Both microinjection and soaking were used to introduce C-QDs into the zebrafish embryos, confirming that the operations are versatile strategies for the introduction of different species into the zebrafish for the study of other drugs and nanomaterials. The fluorescence bioimaging of zebrafish using C-QDs as a probe provides the example that can be applied in basic life science research, toxicity testing, and drug screening with zebrafish as model.

## Methods

### Reagents

Glucose was purchased from Amresco, Shanghai, China. Phenol red solution, paraffin oil, 1-phenyl-2-thioure, 3-aminobenzoate methanesulfonate were obtained from Sigma-Aldrich, Shanghai, China. Ethylenediamine was purchased from Tianjin Chemical Reagent wholesale company, Tianjin, China. High purity nitrogen was obtained from LiuFang high-tech gas plant, Tianjin, China. All solutions were prepared using ultra-purified water (18.25 MΩ cm) from Aquapro Ultra-purified Water System, Chongqing, China.

### Equipments

Transmission electron microscopy (TEM) images were performed with Tecnai G2 F20, FEI Co., USA and operated at an accelerating voltage of 200 kV. X-ray photoelectron spectroscopy (XPS) analysis was performed by a Kratos Axis Ultra DLD spectrometer fitted with a monochromated Al Kα X-ray source (hν 1486.6 eV), hybrid (magnetic/electrostatic) optics, a multichannel plate, and delay line detector. UV-visible absorption spectra were recorded by the UV-2450 spectrophotometer, Shimazu Corporation, Japan. The fluorescence spectra of the C-QDs were performed by FL-4500 fluorescence spectrometer, Hitachi, Japan. The brightfield and fluorescence images were acquired under the Leica 200 M 2000 microscope with DP71 digital camera, Olympus, Japan which mounted on a BX51 fluorescent microscope, Olympus DP71, Japan. The milli-pulse pressure injector used in the experiment was ASI, MPPI-3, USA.

Excitation-dependent emissions were observed when acquiring fluorescence images, which validated the multicolor properties of C-QDs. Sensitivity (ISO 400) was maintained when acquiring fluorescence images of zebrafish and their embryos to reduce interference from the equipment. Brightfield images were obtained to record the morphology of zebrafish embryos or larvae before acquiring fluorescence images. Control samples were used to optimize the exposure time and acquired the nearly invisible fluorescence images. This procedure was repeated every time when acquiring images at different periods.

### C-QDs Synthesis

40 mg of glucose, 10 mL of ultra-purified water, and 100 μL of ethylenediamine were added into the lining (30 mL) of a Teflon-lined autoclave vessel (Fig. S6A).Then the lining was put in an ultrasonic cleaner and ultrasonicated for 10 min to obtain a colorless and transparent solution. The lining sealed in the vessel and heated at 200 °C for 4 h and then naturally cooled to ambient temperature (20 °C–30 °C) (Fig. S6B). The obtained brown solution was centrifuged at 12000 rpm for 10 min. The supernatant was then collected and lyophilized for 28 h to obtain a brown C-QDs paste, which can be dissolved again for later use. A step-to-step procedure for the preparation of C-QDs can be found in SI.

### Zebrafish Husbandry and Embryo Harvesting

Zebrafish were cultured in 10 L aquaria with recycled water (control the salinity to approximately 450–500 μs cm^−1^ with NaCl and NaHCO_3_ and pH 7.0–7.2; add 10% fresh deionized water each day) at 28.5 °C with a 10/14-h dark/light cycle (light on 7:00 A.M.–9:00 P.M.). They were fed with 3 ml of fresh hatched live brine shrimp (hatched from 3.5 g of brine shrimp embryos in 1 L of water) periodically at 8:00 A.M., 12:00 noon and 5:00 P.M every day in every 10 L aquarium. (CAUTION: Zebrafish and their embryos are sensitive to pH and temperature, so make sure that the pH of the medium is 7.0–7.2 and culture them at 28.5 °C; there should be less than 10 adult zebrafish in a 10 L aquarium.) All experimental protocols using animals were approved by the Institutional Animal Care Committee of Nankai University. The methods were carried out in accordance with the approved guidelines.

One mature female zebrafish and two mature male zebrafish were selected and placed in different sides of a 1.5 L breeding cage separated using a divider at 5:30 P.M. (Fig. S7A and B). The divider was removed from the breeding cage at 8:30 A.M. on the next day (Fig. S7C). Half an hour later, the embryos were harvested from the breeding cage by a strainer and raised with E3 medium while the unfertilized embryos and debris were removed (Fig. S7D). (CAUTION: Do not disturb the zebrafish during the spawning time to avoid affecting their spawn.) Zebrafish can be regrouped in larger aquaria to produce additional generations of embryos. A step-to-step procedure for zebrafish husbandry and embryo harvesting can be found in SI.

### Preparation of Microinjection Needles

For preparation of the microinjection needle, 2 μL of 25% phenol red solution was pulled into a needle using a microloader pipette. The needle was inserted into the microinjector and sealed tightly. Turn on the air source and microinjector. Depress the foot pedal and monitor the drop diameter of the phenol red solution while trimming the needle under a microscope and adjusting the injection pressure as required. The drop diameter of the phenol red solution in paraffin oil is used to calculate the volume for a single injection. A bead of phenol red solution with a diameter of 0.12 mm was 1 nL (volume required for a single microinjection) of phenol red solution (Fig. S8A). (CAUTION: The ideal injection volume is approximately 10% of an embryo’s volume, e.g. 1 nL used in this work). The needle was pinched off by a pair of sharp forceps to tat an appropriate point so that the needle can inject 1 nL of C-QDs solution into an embryo and deliver a consistent solution bead size. A step-to-step procedure for preparation of microinjection needles can be found in SI.

### Introduction of C-QDs to Embryos by Soaking

**Figure 7:**
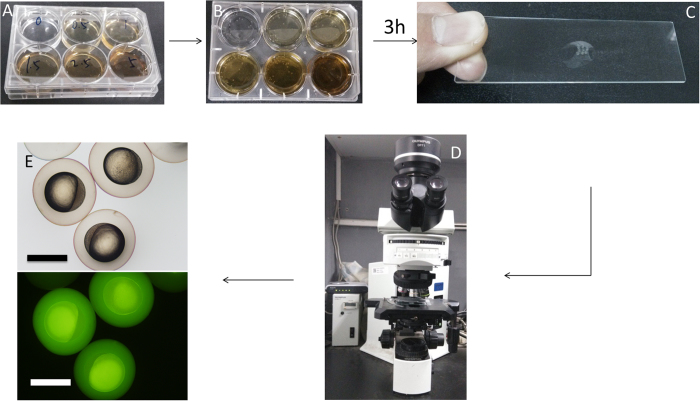
Procedure of introducing C-QDs by soaking and acquiring images of zebrafish embryos. (**A**, **B**) Soaking embryos in C-QDs solutions at different concentrations: 0.5, 1, 1.5, 2.5 and 5 mg mL^−1^. (**C**, **D**) Acquiring images of zebrafish embryos. (**E**) Bright field (upper) and fluorescence (lower) images of zebrafish embryos after soaking with 2.5 mg mL^−1^ of C-QDs. Scale bars, 1.0 mm.

Nanoparticles can enter embryos through endocytosis. Soaking was first used to introduce C-QDs into the zebrafish embryos to investigate the effect on the development of embryos and imaging application of C-QDs. An appropriate amount of C-QDs was dissolved in E3 medium to prepare 5 mL of C-QDs solution of different concentrations. 5 ml of the C-QDs solutions were added to each well of six-well flat-bottom cell culture plates (Fig. 7A). 20 zebrafish embryos were placed in each well of the cell culture plates and soaked with 5 mL of medium that contains C-QDs at different concentrations (Fig. 7B). Before soaking, ensure that the embryos have not developed beyond the four-cell stage, i.e. 1 hour post-fertilization (hpf). Ideally, these embryos should be at the one-cell stage (0.5 hpf) to ensure that C-QDs can permeate into embryos and disperse throughout the embryo. After 3 h, the embryos were rinsed thrice with E3 medium to remove excess C-QDs. The embryos were cultured in E3 medium at 28.5 °C with a 10/14-h dark/light cycle. The culture solution was replaced with E3 medium that contains 0.003 wt.% PTU each day at 8:30 A.M. and 5:30 P.M. to block pigmentation and mediate visualization.

The embryos soaked with C-QDs at different concentrations were placed on the concave side of a single concave glass slide through a dropper and immersed in the E3 medium (Fig. 7C). The glass slide was placed under fluorescence microscope and the embryos were placed into an appropriate position for fluorescence imaging using a fluorescence microscope (Fig. 7D). The bright field and fluorescence images of zebrafish embryos were acquired using a 10× ocular lens and a 4× objective lens (Fig. 7E). The images of embryos with C-QDs at different concentrations were acquired at 3, 6, 12, 24, 48, and 60 hpf. A step-to-step procedure for introduction of C-QDs to zebrafish embryo by soaking can be found in SI.

### Introduction of C-QDs to Embryos by Microinjection

**Figure 8:**
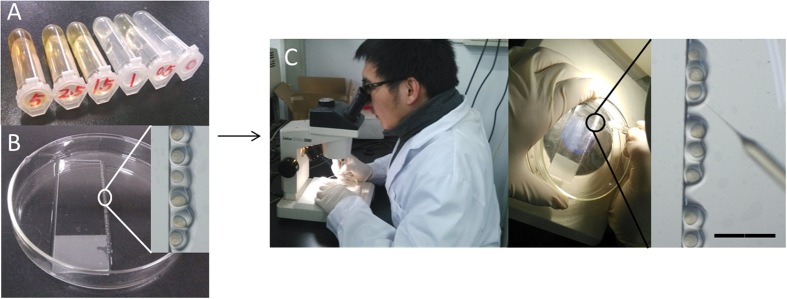
Procedures for microinjection of C-QDs into embryos. (**A**) C-QDs solutions at different concentrations used in microinjection experiment. (**B**) Embryos arranged neatly one by one beside the glass slide in a Petri dish (inset: amplifying image of embryos). (**C**) Microinjection of C-QDs into embryos under a microscope while amplifying embryos and the needle tip. Scale bar: 4.0 mm.

Microinjection has been used for generating transgenic zebrafish after injecting DNA, for gene knockdown after injecting antisense morpholinos, for studying infectious diseases after injecting microbes, and for studying tumor progression after injecting cancer cells^43^. Microinjection of C-QDs was used to illustrate the procedure with the as-prepared microinjector in Fig. S8. 10 mg of C-QDs was dissolved in 2 mL of ultra-purified water to obtain a 5 mg mL^−1^ C-QDs solution and diluted to 2.5, 1.5, 1.0 and 0.5 mg mL^−1^ with ultra-purified water (Fig. 8A). The embryos were lined up against the side of the slide through a transfer pipette to form a single column (Fig. 8B). 2 μL of C-QDs solution was pulled into the needle prepared in advance by a microloader pipette. After Piercing the surface of the chorion and the needle entering into the yolk, 1 nL (bead diameter of 0.12 mm) of the C-QDs solution was injected into the embryos through the microinjector equipment by depressing the foot pedal (Fig. 8C).The solution should be injected into the embryo yolk quickly and accurately so that the embryos survive and grow. Air bubbles should be avoided while the injecting process because they can be lethal for embryos. Before microinjection, ensure that the embryos have not developed beyond the four-cell stage (1 hpf). Ideally, embryos should be at the one-cell stage (0.5 hpf) to ensure that C-QDs will be dispersed in the whole embryo. The injected embryos were moved into clean Petri dishes using a gentle stream of E3 medium immediately after completing a column of embryos and the marked the Petri dishes with the C-QD concentration injected into the embryos. The embryos were cultured in E3 medium at 28.5 °C with a 10/14-h dark/light cycle. The culture solution was replaced with E3 medium that contains 0.003 wt.% PTU at 8:30 A.M. and 5:30 P.M. every day to block pigmentation and mediate visualization. A step-to-step procedure for introduction of C-QDs to zebrafish embryo by microinjection can be found in SI.

### Introduction of C-QDs to Zebrafish Larvae by Soaking

**Figure 9:**
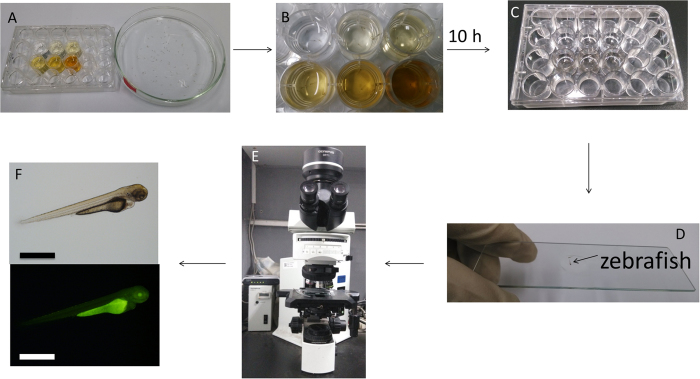
(**A**, **B**) Culture five to seven zebrafish larvae (72 hpf) in C-QDs solution at different concentrations in each of six wells in a 24-well flat-bottom culture plate. (**C**) Soak zebrafish larvae with a 0.016% 3-aminobenzoate methanesulfonate solution to anesthetize them. (**D**) Place the zebrafish larva on the concave side of a single concave glass slide to keep the 3-aminobenzoate methanesulfonate solution immersing the larva. (**E**) Acquire images of zebrafish larva. (**F**) Bright field (upper) and fluorescence images (lower) of zebrafish larva soaked in 5 mg mL^−1^ of C-QDs. Scale bars, 1.0 mm.

The fast development of zebrafish provides the chance to study the distribution of nanomaterials, drug screening, and the study of the toxicity of nanomaterials. The imaging of zebrafish larvae with C-QDs as probe illustrates the operation and procedure. Zebrafish embryos were cultured in E3 medium at 28.5 °C with a 10/14-h dark/light cycle until larvae hatched (approximately 72 hpf). 2 mL of C-QDs solution at different concentrations were prepared (Fig. 9A). 2 mL of culture solution that contains C-QDs at different concentrations and 5–7 zebrafish larvae were added in each well of the 6 wells in a 24-well cell flat-bottom culture plate (Fig. 9B). The larvae were cultured in E3 medium at 28.5 °C with a 10/14-h dark/light cycle. After 10 h, the C-QDs solution was replaced with E3 medium and larvae were washed thrice with E3 medium with a dropper to remove excess C-QDs.

## Additional Information

**How to cite this article**: Kang, Y.-F. *et al.* Carbon Quantum Dots for Zebrafish Fluorescence Imaging. *Sci. Rep.* **5**, 11835; doi: 10.1038/srep11835 (2015).

## Supplementary Material

Supplementary Information
