# Supplementary material for: Carbon Quantum Dots for Zebrafish Fluorescence Imaging
Source: Sci Rep. 2015 Jul 2;5:11835. doi: 10.1038/srep11835 (PMC4488761; doi:10.1038/srep11835)
Supplement: Supplementary Information [file srep11835-s1.doc]

Supporting Information for

Carbon Quantum Dots for Zebrafish Fluorescence Imaging

Yan-Fei Kang ^1^, Yu-Hao Li ^2^,Yang-Wu Fang^2^, Yang Xu^1^, Xiao-Mi Wei^1^, Xue-Bo Yin ^1^,*

^1^ Research Center for Analytical Sciences, College of Chemistry, Nankai University, Tianjin Key laboratory of Biosensing and Molecular Recognition, State Key Laboratory of Medicinal Chemical Biology, Collaborative Innovation Center of Chemical Science and Engineering (Tianjin), Tianjin 300071, China

*Correspondence should be addressed to X.B.Y. E-mail: [xbyin@nankai.edu.cn](mailto:xbyin@nankai.edu.cn); Fax: (+86) 22 23503034

^2^ Tianjin Key Laboratory of Tumor Microenviroment and Neurovascular Regulation, School of Medicine, Nankai University, Tianjin, 300071, China


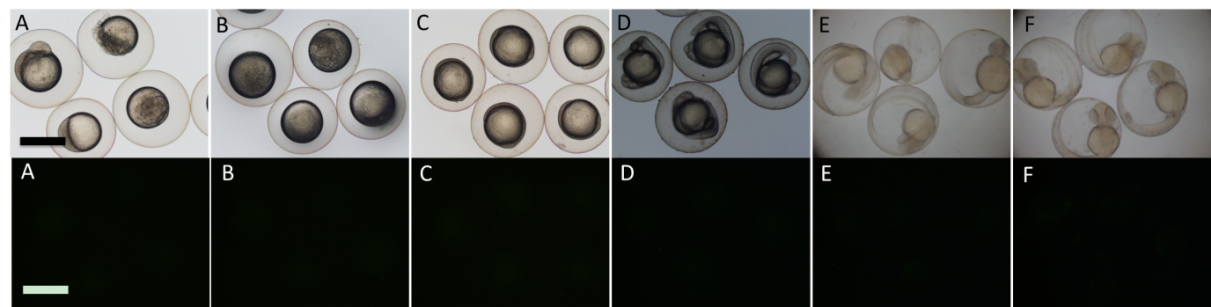


**Figure S1.** Brightfield (upper) and fluorescence (lower) images of zebrafish embryos after culturing in E3 culture solution for 3 h at different time points: (A) 3, (B) 6, (C) 12, (D) 24, (E) 48, (F) 60 hpf. A 10× ocular lens and a 4× objective lens were used. Scale bars, 1.0 mm.


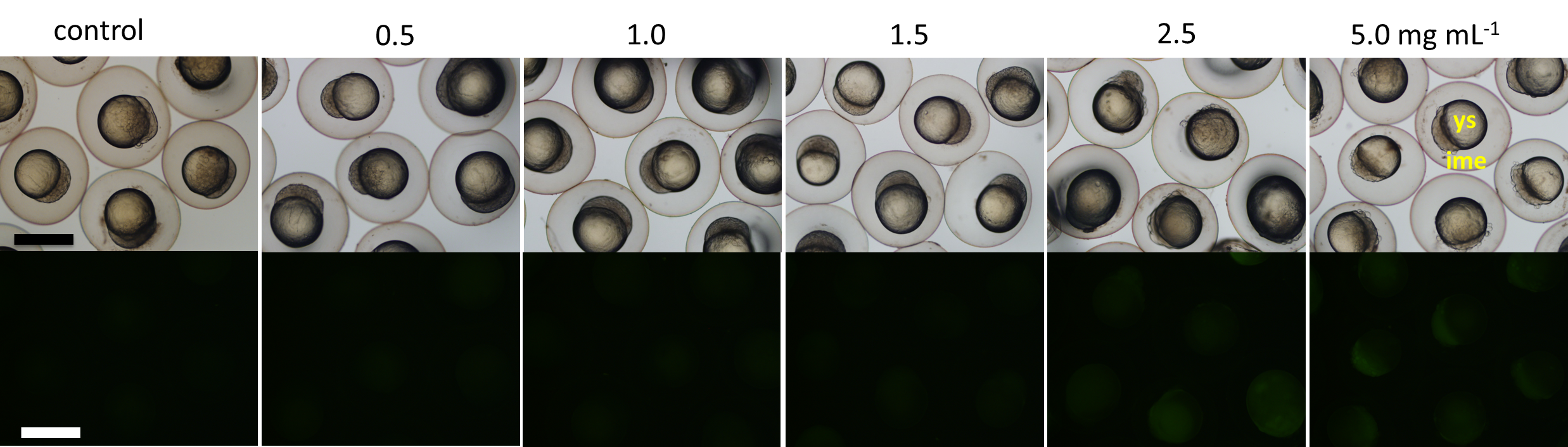


**Figure S2.** Bright field (upper) and fluorescence (lower) images of zebrafish embryos at 3 hpf after being injected with 1 nL of C-QDs solutions at different concentrations. Images acquired under bright light show the yolk sac (ys) and the inner mass of embryos (ime). A 10× ocular lens and a 4× objective lens were used. Scale bars, 1.0 mm.


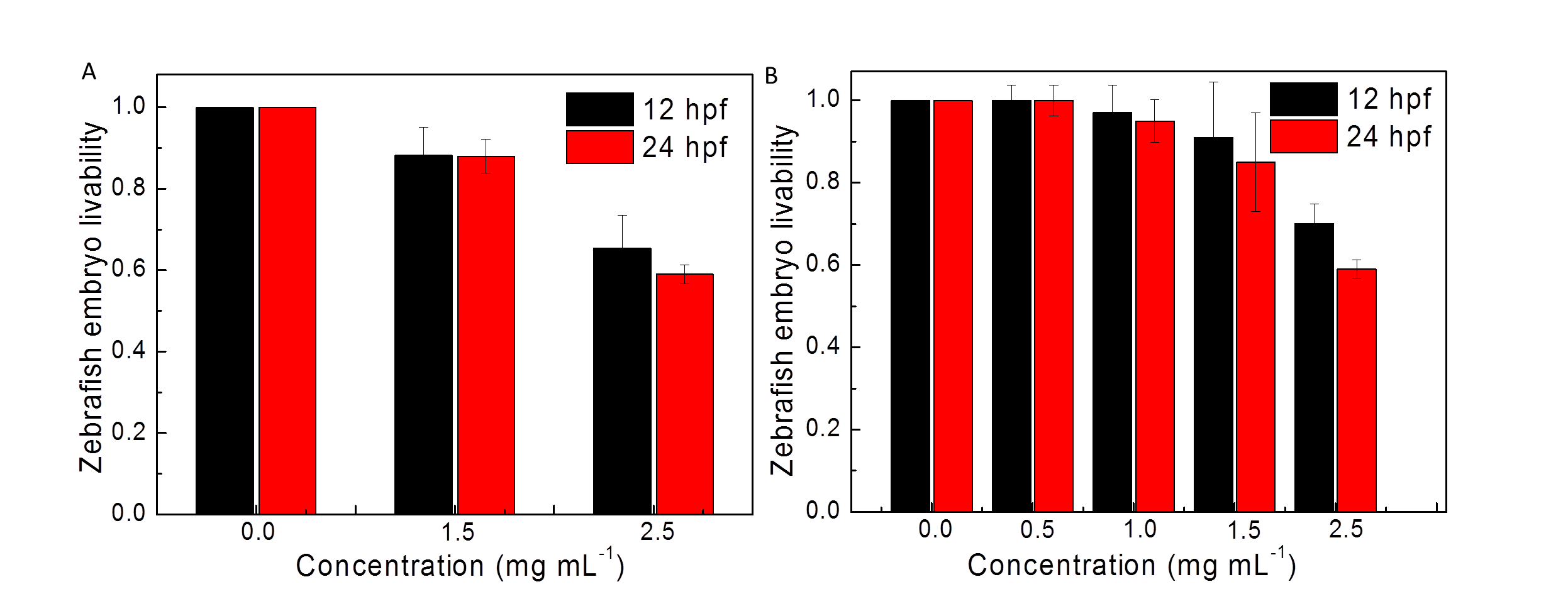


**Figure S3.** (A) Effect of the C-QDs with different concentrations on the livability of zebrafish embryos at 12 hpf and 24 hpf after microinjected with C-QDs solutions during 0-3 hpf. (B) Effect of the C-QDs with different concentrations on the livability of zebrafish embryos at 12 hpf and 24 hpf after soaking in C-QDs solutions during 0-3 hpf. Data presented as mean ±SD (n=3).

**
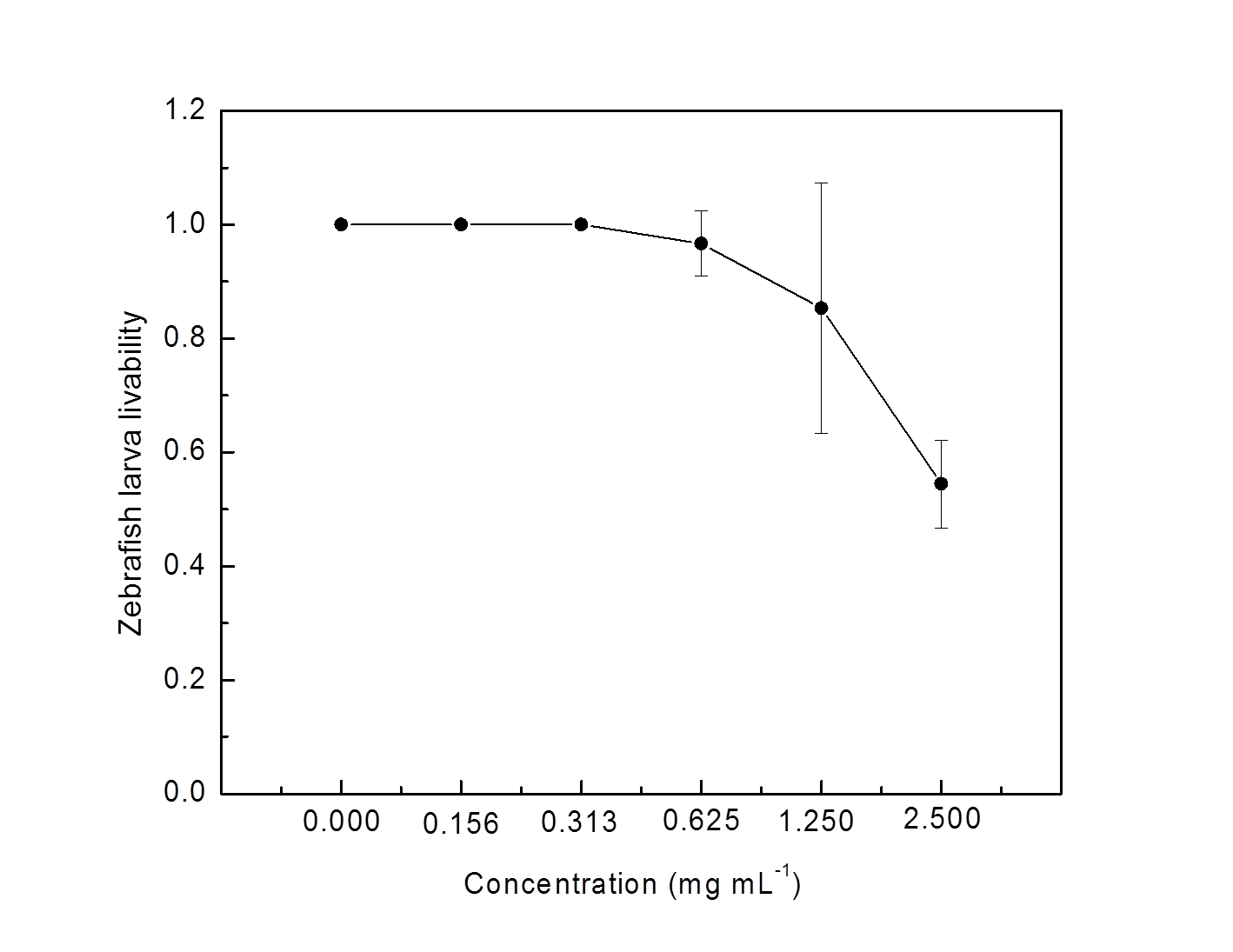
**

**Figure S4.** Effect of the C-QDs with different concentrations on the livability of zebrafish larvae at 84 hpf after soaking in C-QDs solutions for 10 h. Data presented as mean ±SD.


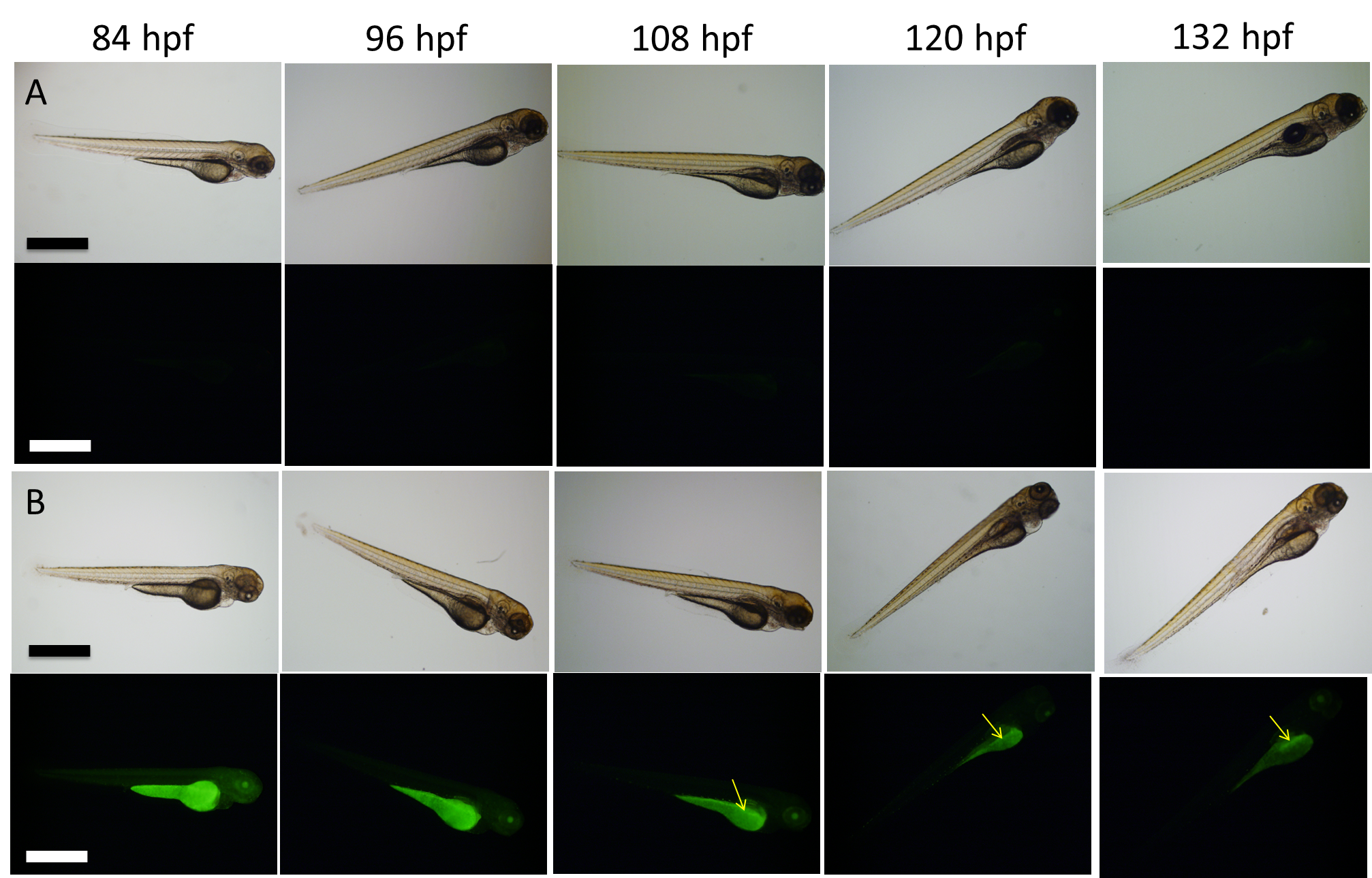


**Figure S5.** Brightfield (upper) and fluorescence (lower) images of zebrafish larva at different time point after incubating in E3 medium (A) and 1.5 mg mL^-1^ C-QDs solution (B) during the period from 74 to 84 hpf. Yellow arrows: intestine. Scale bars, 1.0 mm.

**The step-by-step procedure of the main operations in the experiment**

**1. C-QDs** **synthesis**

1. Add 40 mg of glucose, 10 mL of ultra-purified water, and 100 μL of ethylenediamine into the lining (30 mL) of a Teflon-lined autoclave vessel (Figure S6A).

2. Place the lining in an ultrasonic cleaner and ultrasonicate the lining for 10 min to obtain a colorless and transparent solution. The water level in the ultrasonic cleaner should be higher than that inside the linings to ensure that the glucose and ethylenediamine are dissolved in water. Be careful that the water in the ultrasonic cleaner does not contaminate the mixture.

3. Place the lining into the autoclave vessel. After sealing, the autoclave vessel is placed into an electro-thermostatic blast oven and heated at 200 °C for 4 h (Figure S6B). Four parallel tests are simultaneously performed to obtain sufficient C-QDs. Turn on the blast so that the temperature is uniform in the oven.

4. Take the autoclave vessels out of the oven and allow them to cool to ambient temperature (20 °C–30 °C) after heating for 4 h. Wear oven gloves when removing the autoclave vessels from the oven to prevent burns.

5. Centrifuge the brown solution at 12000 rpm for 10 min and collect the supernatant in a clean Petri dish.

6. Place this Petri dish in a freezer (−20 °C) until the solution is frozen.

7. Lyophilize the frozen product for 28 h to obtain a brown C-QDs paste, which can be dissolved again for later use.


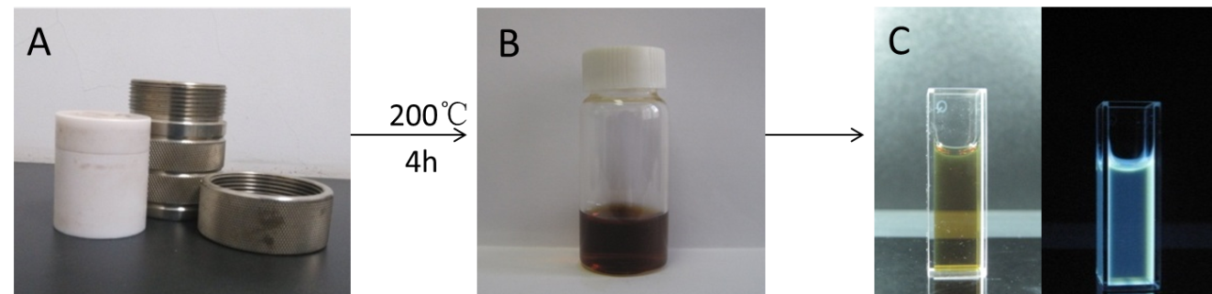


**Figure S6**. Procedures for synthesizing C-QDs and photos of C-QDs solution with and without excitation. (A) Autoclave vessel and lining, (B) Original C-QD solution, and (C) Photos of C-QDs solution without (left) and with (right) UV light excited at 365 nm.

**2. Zebrafish husbandry and** **embryo** **harvesting**

1. Culture zebrafish in aquaria with recycled water (control the salinity to approximately 450–500 μs cm^-1^ with NaCl and NaHCO_3_; pH 7, and add 10% fresh deionized water each day) at 28.5 °C with a 10/14-h dark/light cycle (light on 7:00 A.M.–9:00 P.M.), which is controlled with a machine. Feed zebrafish with 3 ml of fresh hatched live brine shrimp (obtained from 3.5 g of brine shrimp embryos in 1 L of water) in each 10 L aquarium periodically at 8:00 A.M., 12:00 noon and 5:00 P.M. each day. (All experimental protocols using animals were approved by the Institutional Animal Care Committee of Nankai University. The methods were carried out in accordance with the approved guidelines.)

2. Select one mature female zebrafish and two mature male zebrafish and place them in different sides of a 1.5 L breeding cage using a divider at 5:30 P.M. (Figure S7A and B)Using a breeding cage is necessary because zebrafish may eat their embryos if they come into touch with the embryos. Culture four to five breeding cages zebrafish to obtain sufficient embryos simultaneously.

3. Remove the divider from the breeding cage at 8:30 A.M. on the next day and allow approximately 30 min of undisturbed mating time. Do not disturb the zebrafish during the spawning time to avoid affecting their spawning (Figure S7C).

4. Harvest the embryos from the breeding cage with a strainer half an hour later and rinse them with E3 medium. Pour embryos into a Petri dish that contains E3 medium and remove unfertilized embryos and debris with a transfer pipette (Figure S7D).

5. Zebrafish can be regrouped in larger aquaria to produce additional generations of embryos.

**
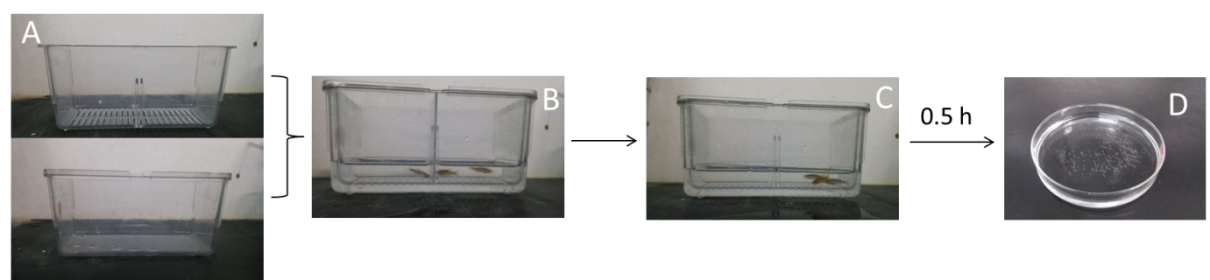
**

**Figure S7.** Procedures of zebrafish embryo harvesting. (A) Breeding cage. (B) Culturing one mature female zebrafish and two mature male zebrafish in different sides of a 1.5 L breeding cage separated with a divider in place. (C) Removing the divider from the breeding cage for spawning. (D) Harvesting the embryos from the bottom of the breeding cage with a strainer and place them into clean Petri dishes with E3 medium half an hour later.

**3. Preparation of microinjection needles**

1. Pull 2 μL of a 25% phenol red solution (the color of the phenol red solution is conspicuous, Figure S8A) into a needle (pulled by a micropipette puller in advance) using a microloader pipette. Inject the phenol red solution in this needle to its tip until no bubbles remain in the needle.

2. Insert the needle into the microinjector and seal it tightly.

3. Turn on the air source and microinjector. Bring the needle tip into the plane of view of a microscope and focus on the thinnest region of the tip.

4. Depress the foot pedal and monitor the drop diameter of the phenol red solution while trimming the needle and adjusting the injection pressure as required. The drop diameter of the phenol red solution in paraffin oil can be used to calculate the volume for a single injection. A bead of phenol red solution with a diameter of 0.12 mm contains 1 nL (volume required for a single injection) of phenol red solution (Figure S8A).

5. Use a pair of sharp forceps to pinch off the needle at an appropriate point so that the needle will inject 1 nL of C-QDs solution into an embryo and deliver a consistent solution bead size. The ideal mciroinjection volume is approximately 10% of an embryo’s volume (e.g. 1 nL used in this protocol).


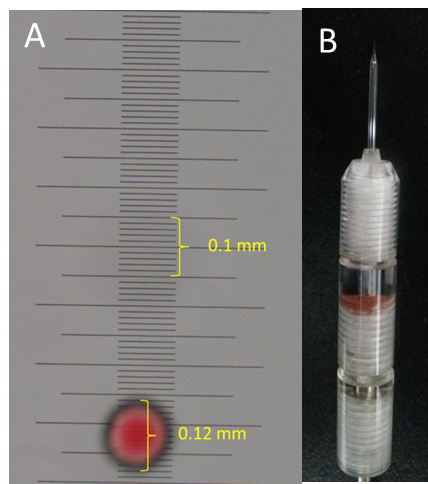


**Figure S8.** (A) 1 nL of phenol red solution in paraffin oil has a diameter of 0.12 mm. (B) Microinjection needle.

**4. Microjecting C-DQs into embryos**

1. Dissolve 10 mg of C-QDs in 2 mL of ultra-purified water to obtain a 5 mg mL^-1^ C-QDs solution. Dilute this solution to 2.5, 1.5, 1.0 and 0.5 mg mL^-1^ with ultra-purified water (Figure 7A).

2. Place a microscope slide in a 90-mm Petri dish and use a transfer pipette to line up the embryos against the side of the slide to form a single column. Remove excess E3 medium from the slide (Figure 7B). Be certain that all embryos are soaked with E3 medium so that they do not dehydrate and die.

3. Using the needle prepared in advance, place 2 μL of the as-prepared C-QDs solution into the needle using a microloader pipette and remove air bubbles.

4. Turn on the air source and microinjector. Insert the needle into the microinjector and seal it tightly.

5. Pierce the surface of the chorion and enter the yolk with the needle.

6. Depress the foot pedal and inject 1 nL (bead diameter of 0.12 mm) of the C-QDs solution at different concentrations [control (0), 0.5, 1, 1.5, 2.5 and 5 mg mL^-1^] into the embryos through the microinjector equipment (Figure 7C). Inject approximately 50 embryos with each C-QD solution concentration to ensure there are sufficient surviving embryos. Solution should be injected into the embryo yolk sac quickly and accurately so that the embryos survive and grow. Avoid injecting air bubbles into embryos because this can be lethal for embryos. (Before microinjection, ensure that the embryos have not developed beyond the four-cell stage (1 hpf). Ideally, embryos should be at the one-cell stage (0.5 hpf) to ensure that C-QDs will be dispersed in the whole embryo.)

7. Move the injected embryos into clean Petri dishes using a gentle stream of E3 medium immediately after completing a column of embryos and mark the Petri dishes with the C-QD concentration injected into the embryos.

8. Culture these embryos in E3 medium at 28.5 °C with a 10/14-h dark/light cycle. Replace the culture solution at 8:30 A.M. and 5:30 P.M. each day with E3 medium that contains 0.003 wt.% PTU to block pigmentation and mediate visualization. The time of adding PTU is flexible; however, it must be added before 24 hpf.

**5. Introducing C-DQs into embryos by soaking**

1. Dissolve an appropriate amount of C-QDs in E3 medium to prepare 5 mL of C-QD solution of different concentrations: 0.5, 1, 1.5, 2.5 and 5 mg mL^−1^. Add 5 ml of these C-QD solutions to each well of six-well flat-bottom cell culture plates (Figure 6A). All C-QDs solutions should be prepared using E3 medium.

2. Collect embryos in a Petri dish with E3 medium. Place 20 zebrafish embryos in each well of six-well flat-bottom cell culture plates and then soak the embryos with 5 mL of medium that contains C-QDs at different concentrations: control (0), 0.5, 1, 1.5, 2.5 and 5 mg mL^−1^ (Figure 6B). (Before this procedure, ensure that the embryos have not developed beyond the four-cell stage (1 hpf). Ideally, these embryos should be at the one-cell stage (0.5 hpf) to ensure that C-QDs can permeate into embryos and will disperse throughout the embryo.)

3. After 3 h, rinse the embryos thrice with E3 medium to remove excess C-QDs. Remove the unfertilized embryos and debris during this procedure.

4. Culture the embryos with E3 medium at 28.5 °C with a 10/14-h dark/light cycle. Replace the culture solution each day at 8:30 A.M. and 5:30 P.M. with E3 medium that contains 0.003 wt.% PTU to block pigmentation and mediate visualization. (The time for adding PTU is flexible; however, it must be added before 24 hpf.)

**6. Fluorescence imaging of** **zebrafish embryos**

1. Place the embryos that were injected or soaked with C-QDs of different concentrations on the concave side of a single concave glass slide using a dropper and use E3 medium to immerse the embryos (Figure 6C).

2. Place the glass slide under a fluorescence microscope and manipulate embryos into an appropriate position (Figure 6D).

3. Fluorescence imaging is performed using a fluorescence microscope. Acquire bright field and fluorescence images of zebrafish embryos using a 10× ocular lens and a 4× objective lens (Figure 6E).

4. Acquire images of embryos injected/soaked with C-QDs at different concentrations under white light and with excitation at 460–495 nm at 3, 6, 12, 24, 48, and 60 hpf.

5. Anesthetize embryos with 0.016% 3-aminobenzoate methanesulfonate solution (sufficient to soak the embryos) before acquiring images in case of their movement during the period of acquiring images.

6. After imaging, embryos are put back in E3 medium in six-well flat-bottom cell culture plates and they will revive.

**7. Soaking zebrafish larvae to introduce C-DQs**

1. Culture zebrafish embryos with E3 medium at 28.5 °C with a 10/14-h dark/light cycle. Replace their culture solution at 5:30 P.M. with E3 medium that contains 0.003 wt.% PTU to block pigmentation and mediate visualization. Replace the medium with E3 medium that contains 0.003 wt.% PTU each day at 8:30 A.M. and 5:30 P.M. until larvae hatch (approximately 72 hpf). The time for adding PTU is flexible; however, it must be added before 24 hpf.

2. Prepare 2 mL of C-QDs solution at different concentrations: 0.156, 0.312, 0.620, 1.25 and 2.50 mg mL^-1^ (Figure 8A).

3. Select six wells from a 24-well cell flat-bottom culture plate. Add 2 mL of culture solution that contains C-QDs at different concentrations and 5-7 zebrafish larvae in each well (Figure 8B).

4. 10 h later, replace the C-QDs solution with E3 medium and wash larvae thrice with E3 medium with a dropper to remove excess C-QDs. Remove dead larvae and debris during this procedure.

**8. Fluorescence imaging of zebrafish larvae**

1. Anesthetize larvae with 3 mL of 0.016% 3-aminobenzoate methanesulfonate solution before acquiring images in case of their movement during the period of acquiring images (Figure 8C).

2. Place the larvae soaked with C-QD solution on the concave side of a single concave glass slide using a dropper while immersing the larvae in the 3-aminobenzoate methanesulfonate solution (Figure 8D). Ensure that larvae are soaked in the solution when acquiring images so that they do not die.

3. Place the glass slide under a fluorescence microscope and acquire fluorescence images. Acquire bright field and fluorescence images for the same part of a zebrafish larva using different objective lens (Figure 8E).

4. Acquire images of whole zebrafish larva using a 10× ocular lens and a 4× objective lens. Acquire magnified images of a part of larva, such as eyes, yolk sac and tail, using a 10× ocular lens and a 10× objective lens (Figure 8F). Adjust for the appropriate exposure time (described in equipment section) and acquire fluorescence images for zebrafish larva with C-QDs introduced at different concentration using the same exposure time.

5. After imaging, zebrafish are put back in E3 medium in six-well flat-bottom cell culture plates and they can revive. At 9 A.M. and 6 P.M. in each day (from 5 dpf), change the zebrafish medium with the E3 medium contains a certain amount of the paramecia and larvae are fed with paramecia (paramecia are cultured in distilled water).
